# Effect of *ACTN3* Genotype on Sports Performance, Exercise-Induced Muscle Damage, and Injury Epidemiology

**DOI:** 10.3390/sports8070099

**Published:** 2020-07-13

**Authors:** Gabriel Baltazar-Martins, Jorge Gutiérrez-Hellín, Millán Aguilar-Navarro, Carlos Ruiz-Moreno, Victor Moreno-Pérez, Álvaro López-Samanes, Raúl Domínguez, Juan Del Coso

**Affiliations:** 1 Exercise Physiology Laboratory, Camilo José Cela University, 28692 Madrid, Spain; jgsoares@ucjc.edu (G.B.-M.); millan.aguilar@ufv.es (M.A.-N.); cruizm@ucjc.edu (C.R.-M.); 2 Faculty of Health Sciences, Universidad Francisco de Vitoria, 28223 Madrid, Spain; jghuniversidad@gmail.com (J.G.-H.); alvaro.lopez@ufv.es (Á.L.-S.); 3 Centre for Translational Research in Physiotherapy, Universidad Miguel Hernández, 03202 Elche, Spain; vmoreno@umh.es; 4 College of Health Sciences, Isabel I University, 09003 Burgos, Spain; raul_dominguez_herrera@hotmail.com; 5 Centre for Sport Studies, Rey Juan Carlos University, 28943 Fuenlabrada, Spain

**Keywords:** genetics, exercise performance, elite athlete, injury risk, sports performance, muscle power

## Abstract

Genetic factors play a significant role in athletic performance and its related phenotypes such as power, strength and aerobic capacity. In this regard, the lack of a muscle protein due to a genetic polymorphism has been found to affect sport performance in a wide variety of ways. α-actinin-3 is a protein located within the skeletal muscle with a key role in the production of sarcomeric force. A common stop-codon polymorphism (rs1815739; R577X) in the gene that codes for α-actinin-3 (*ACTN3*) produces individuals with the XX genotype that lack expression of a functional α-actinin-3. In contrast, individuals with the R-allele (i.e., RX vs. RR genotypes) in this polymorphism can express α-actinin-3. Interestingly, around ~18% of the world population have the XX genotype and much has been debated about why a polymorphism that produces a lack of a muscle protein has endured natural selection. Several investigations have found that α-actinin-3 deficiency due to XX homozygosity in the *ACTN3* R577X polymorphism can negatively affect sports performance through several structural, metabolic, or signaling changes. In addition, new evidence suggests that α-actinin-3 deficiency may also impact sports performance through indirect factors such a higher risk for injury or lower resistance to muscle-damaging exercise. The purpose of this discussion is to provide a clear explanation of the effect of α-actinin-3 deficiency due to the *ACTN3* XX genotype on sport. Key focus has been provided about the effect of α-actinin-3 deficiency on morphologic changes in skeletal muscle, on the low frequency of XX athletes in some athletic disciplines, and on injury epidemiology.

## 1. Introduction

Athletic performance is a complex trait influenced by both heredity (e.g., sex, genetics, and epigenetics) and the environment (e.g., training, diet, and sociodemographic factors). Traditionally, there has been a view that concedes a priority influence of environmental factors to achieve the status of elite athlete, although it seems that some athletes are naturally talented for certain sport disciplines. In this regard, in recent years, evidence suggesting that genetics plays a significant role in athletic performance has increased exponentially and the influences of “nature” (genetics) and “nurture” (environment) on the ability to excel in sport are well recognized [1]. The effect of genetics has been associated to phenotypes such as muscle power, strength, and aerobic capacity that may directly affect physical performance in a myriad of sport disciplines. Although the true influence of genetics on the success of most sports is still unknown, some investigations have found the influence of hereditary factors on the maximal aerobic capacity may reach up to ~50% [2,3]. Hence, “nature” and “nurture” are both necessary factors for elite sporting performance while the complex interaction these factors may mold a talented athlete into a champion [4].

Research in the last few decades has primarily focused on trying to understand the link between certain gene polymorphisms and several aspects of exercise and sports performance. In this regard, more than one hundred candidate genes have been identified as potential contributors to athletic performance [5], but one in particular has been receiving scientific attention—the *ACTN3* gene [6]. This gene encodes the protein α-actinin-3 [7], a bundling protein located within the skeletal muscle with a key role in the production of sarcomeric force. The *ACTN3* gene has attracted the attention of exercise physiologists and sport practitioners because it predetermines the expression of a protein that cross-links and stabilizes actin thin filaments at the Z-disc and hence, is fundamental for the production of forceful contractions and fast and explosive movements [8]. α-actinin-3 expression is found only in the fast type II fibers while there is an isoform of α-actinin-3 (i.e., α-actinin-2; 81% identical and 91% similar [9]) that is ubiquitously expressed in all muscle fiber types [10]. Although α-actinin-2 and -3 are almost identical and evolved from repeated gene duplication events, it has been speculated that they possess different roles within skeletal muscle [11]. 

In 1996 [12,13], North and co-workers identified a single nucleotide polymorphism in the *ACTN3* gene that negatively affected the expression of α-actinine-3. Specifically, the p.R577X single nucleotide polymorphism (rs1815739) results in the replacement of an arginine (R) with a premature stop codon (X). As a result, individuals who are homozygous for this stop codon in the *ACTN3* gene (i.e., the ones who possess the 577XX genotype, also known as *ACTN3* XX individuals) suffer from α-actinin-3 deficiency. On the contrary, individuals with RX or RR genotypes express a functional α-actinin-3. Moreover, it has been proposed that the *ACTN3* genotype controls the sarcomeric composition and muscle function in a dose-dependent fashion, indicating that RR individuals are the ones with a higher amount of α-actinine-3 within the muscle, even when compared to RX individuals [14]. Although α-actinin-3 deficiency is compensated for by a higher expression of α-actinin-2, the fast type II fibers, scientific evidence has shown that the *ACTN3* XX genotype is underrepresented in elite power-oriented athletes [9], while it may be related to several phenotypes associated to a higher predisposition to sport-related injury [15]. Thus, the *ACTN3* XX and the subsequent deficiency of α-actinin-3 may be indicative of a negative effect of this genetic variation on the function of fast-twitch muscle fibers. The specialized expression pattern of α-actinin-3 in type II muscle fibers and the different allele frequency across different populations of elite athletes points to the fact that the role of 𝛼-actinin-3 cannot be compensated for by a higher expression of 𝛼-actinin-2 in XX individuals.

Interestingly, around ~20% of the world population have the XX genotype [10] and the frequency of XX individuals fluctuates among different geographical locations (~25% in Asians, 18% in Caucasians, 11% in Ethiopians, 3% in US African Americans, and only 1% in Kenyans [16,17,18]). The lack of α-actinin-3 does not produce any disease and has little clinical significance [18] but it is somewhat surprising that this polymorphism has surpassed natural selection during human evolution. The widespread absence of 𝛼-actinin-3 in almost one fifth of the world population suggests that the role of α-actinin-3 in skeletal muscle may be redundant and it is not key for survival. In this regard, α-actinin-3 deficiency has been related to positive phenotypes and this would explain the perpetuation of the *ACTN3* XX genotype through human evolution. In particular, it has been proposed that XX produces an increased metabolic efficiency [19] and it may even give a certain ‘survival’ advantage [20]. The purpose of this discussion is to provide a visual explanation of the effects of α-actinin-3 deficiency on sports performance, on morphologic changes in skeletal muscle, and on the predisposition to some sport-related injuries.

## 2. Effect of α-Actinin-3 Deficiency in Sports Performance

Early evidence on the *ACTN3* XX genotype showed that this particular genotype was underrepresented in international-level athletes of sprint and power-based disciplines, when compared to healthy untrained individuals [21]. This finding has been replicated in subsequent case-control studies showing a higher frequency of the R-allele and particularly of the RR genotype in sprint- and power-based sports [9,22,23,24,25,26]. Interestingly, the higher frequency of athletes with the RR genotype or carrying the R-allele has not been found in power-based sports that require intermittent actions with a technical component for success [27,28], although this is not always the case [29,30]. These data may indicate that the presence of α-actinin-3 due to the *ACTN3* RR genotype may be more important for sports with a “pure” physical component of sprint/power/strength performance, while this genotype may be less important in skill-based sports even in those where power/strength are associated to performance.

All these recently-summarized studies [9] demonstrate that sprint-/power-based athletes across different sports and ethnic backgrounds have lower frequencies of the *ACTN3* XX genotype compared with a population of control individuals. This outcome suggests that the *ACTN3* XX genotype and the deficiency of α-actinin-3 is somewhat unfavorable for fast and powerful exercise efforts, limiting then the likelihood of becoming an elite athlete in this type of sport discipline. For this reason, the *ACTN3* gene has received the name “speed gene” [9] and the RR genotype has been deemed to be a potentially-favorable genetic variation allowing athletes to excel in sprint-like disciplines [31]. This is borne out by the fact that a very high frequency of R-allele carriers was found in samples of sprinters holding world records in sprint races [16] which may be one of the reason for their superiority in running sprint events.

Other evidence from non-elite populations has found that RR individuals present higher strength values [32,33] and higher muscle volume [33,34], while a higher response to strength training has been found in RR than in XX individuals [35]. While muscle fiber composition seems unaffected by α-actinin-3 deficiency [36], the cross-sectional area of type II muscle fibers might be larger in RR than in XX individuals [37]. Regarding the influence of the different *ACTN3* genotypes on training adaptations, greater increases in muscle power after strength training have been found in RR individuals compared to XX individuals [38] while a reduced exercise-induced muscle protein synthesis has also been found in α-actinin-3-deficient individuals [36]. In addition, it appears that the XX genotype has been associated with an increased response to low-intensity resistance training and endurance training [39], while the *ACTN3* RR-genotype carriers display a greater improvement of performance parameters in response to high-intensity resistance training [40]. All this information with non-elite populations suggests that the higher capacity of RR individuals to perform in speed-/power-based sport disciplines might be tied to a better response to strength and power-oriented training [18]. Still, it is unknown if the use of genetic-specific protocols of strength- and power-oriented training may help to offset the differences in anaerobic-like performance between *ACTN3* genotypes. Since there are multiple factors underpinning success in sports and trainability remains one of the main conditioning factors leading to increased performance, future investigations should be aimed at determining if appropriate exercise training is capable of offsetting the potential negative impact of the XX genotype on sprint-/power-based disciplines. Hence, the influence of training on the likelihood of being an elite athlete in sprint disciplines when possessing the XX genotype is an interesting point for future investigation. By performing this research applied to each sports setting, we might be contributing to providing data relevant for various sports and thus improving knowledge of an athlete’s progression.

Regarding endurance performance, an inverse association was initially suggested for endurance-like sport disciplines because a higher frequency of the *ACTN3* XX genotype was found in endurance athletes [21]. This association would explain in part the maintenance of the XX genotype through natural selection and the relatively high frequency of the XX genotype in human populations living in cold environments [41,42]. However, the higher frequency of XX athletes has not been replicated in more recent investigations with cohorts of elite endurance athletes, some of these being the 10,000 m and marathon runners participating in national/international track-and-field championships [22,43] and the ironman triathlon finishers [44]. This lack of association of the XX genotype with endurance performance appears to be evident by the lack of association between *ACTN3* R577X genotype and elite runners’ personal best 1500, 3000, 5000 m and marathon running times [45]. Thus, the current notion indicates that the lack of α-actinin-3 does not offer any advantage nor detrimental effect on endurance performance. Still, it remains controversial as to why the XX genotype is so prevalent in some populations and it is possible that future research may unveil any positive effect of the lack of α-actinin-3 on sports performance.

In summary, given the fact that the RR genotype is often overrepresented in elite sprinters and power-based athletes, in comparison to control populations of untrained individuals, it is highly possible that possessing this genotype favors the options of being an elite athlete in such sport disciplines. Additionally, the lower incidence of certain types of injuries alongside with the reduced markers of exercise-induced muscle damage observed in RR athletes, as discussed in the following sections of this manuscript, could offer an important support for athletic performance. However, the data included in this manuscript do not dispute the presence of other hereditary factors and the necessity of optimal ambient conditions to excel in sport, even in individuals with the RR genotype. It is important to highlight that α-actinin-3 deficiency due to the XX genotype does not preclude the possibility of achieving high performance in power-like sports because other cohorts of elite power/sprint athletes have reported normal frequencies of athletes with the XX genotype [46,47]. This is particularly important in avoiding the misleading information that some direct-to-consumer genetic testing offers regarding the prediction of talent based on the measurement of only the *ACTN3* genotype [48].

## 3. Mice with An Artificially-Induced α-Actinin-3 Deficiency

To explore the effects of 𝛼-actinin-3 deficiency in humans due to the *ACTN3* XX genotype, and to enhance the mechanistic explanation of this deficiency within the skeletal muscle, mice with an artificially-induced 𝛼-actinin-3 deficiency have been developed. This animal model is known as the *Actn3* knockout (KO) mice [49] and consists of a generation of genetically-modified mice that do not express any detectable α-actinin-3 protein. Data obtained from *Actn3* KO animals indicates that α-actinin-3-deficient mice have lower force production and lower weight and lean body mass than mice with normal expression of 𝛼-actinin-3 (i.e., wildtype littermates [50]). Moreover, *Actn3* KO mice have a reduction in fast type II muscle fiber size, a shift towards a slow-twitch aerobic metabolic phenotype, and increased glycogen storage and mitochondrial oxidative enzyme activity [51,52]. These muscle changes found in *Actn3* KO mice point towards an enhanced capacity for fat/carbohydrate oxidation during prolonged exercise that would enhance the obtaining of energy through aerobic metabolic pathways during endurance-based muscle contraction. In fact, these beneficial phenotypes would explain why 𝛼-actinin-3 deficiency due to the *ACTN3* XX genotype has overcome natural selection. However, as mentioned above, the potential advantage of α-actinin-3 deficiency for endurance sports is not clear and some of the findings with the *Actn3* KO model have not been replicated in humans [53].

Another interesting observation was an increased calcineurin activity in *Actn3* KO mice. Calcineurin is a serine/threonine phosphatase resulting in the expression of a set of genes involved in the maintenance, growth, and remodeling of skeletal muscle [54] and mitochondrial biogenesis [55]. Specifically, calcineurin is implicated in fiber-type transformations [56], while it selectively up-regulates the expression of genes associated to slow-twitch muscle fibers. This phenotype found in *Actn3* KO mice could theoretically predispose individuals with α-actinin-3 deficiency to enhanced adaptation to endurance exercise stimuli [57], while pointing toward an unfavorable physiological adaptability of *Actn3* KO mice to strength and power training stimuli [50]. However, the reach of these findings in the animal model has to be tested in humans. In fact, the higher endurance capacity found in *Actn3* KO mice and the mechanism that support this phenotype have to be replicated in humans [9]. More research is still required to demonstrate if XX athletes would also display an increased calcineurin activity, resulting in a favorable endurance-like phenotype. 

The shift in muscle function due to 𝛼-actinin-3 deficiency remains to be fully explained, but the *Actn3* KO mouse model has helped to determine changes in structural, metabolic, signaling, and calcium handling processes that explain the results found in humans. Based on the data obtained with this animal model, the muscle tissue of α-actinin-3-deficient individuals is potentially more prone to obtaining adaptations from endurance training rather than from strength- or power-oriented training although such speculation has to be confirmed by further investigation in humans beyond the current evidence [18,53].

## 4. Effect of α-Actinin-3 Deficiency on Injury Epidemiology

There is some developing evidence regarding the influence of the *ACTN3* R577X genotype on the incidence of exercise-related muscle injury, although outcomes are unclear. Initially, a study with female athletes suggested that R-allele carriers have a higher probability of suffering non-contact muscle injuries while practicing different sports activities when compared to X-allele carriers [58]. On the contrary, a study with first-division soccer players displayed that those players with the XX genotype had a threefold higher likelihood of suffering muscle injuries [59]. Since these two initial investigations with distinct outcomes, the evidence pointing to XX athletes being more prone to suffering sports-related muscle injuries when compared to RR counterparts has evolved, confirming, in most cases, the negative role of the *ACTN3* XX genotype on non-contact muscle injuries in cohorts of elite performance players [60] and amateur marathon runners [15]. In these investigations, besides the injury incidence, XX athletes had a higher frequency of sudden-onset injuries [15] and the injuries reported needed more days for full recovery [59]. Although the mechanism to explain the likely higher incidence of sports-related muscle injury in XX athletes is yet to be found, it may be speculated that the lack of α-actinin-3 reduces the capacity of the skeletal muscle to endure the exercise contractions that lead to muscle injury. As indicated above, α-actinin-3 is an actin-binding protein with a key role in anchoring actin filaments to the Z-line. The lack of α-actinin-3 in fast-type muscle fibers, due to the *ACTN3* XX genotype, would produce, then, a less powerful link between the actin filaments and the Z-line which may result in a structural deficiency that leads to a sarcomere more prone to suffering damage under high mechanical stress. Whether the implementation of strength training programs can be useful to counteract the potentially higher predisposition for exercise-associated muscle injury observed in XX athletes remains to be studied in future investigations. Therefore, more investigation is warranted to determine if proper strength-based training, or other forms of training such as stretching, may counteract the increased tendency for muscle injury during exercise in XX athletes. To date, whether determining the XX athletes may reduce the impact of this genotype on any aspect of sports performance due to exercise, is speculation due to the low number of investigations on this topic [18].

There is greater consensus on the higher predisposition of XX individuals to suffer ankle sprains during daily activities [61,62,63]. This finding has been replicated in samples of patients with acute and non-acute ankle sprain, but who are otherwise healthy individuals [62,63] and in ballerinas [61] but there is more evidence to depict the effect of the *ACTN3* genotype of ligament injuries in sports. Finally, it has been found that women with the XX genotype have lower bone mineral density at several body locations than their RR counterparts [64]. This same finding has also been reported in the *Actn3* KO mouse model [65]. In men, it has been shown that the XX genotype might be associated with higher serum levels of osteocalcin, a protein hormone produced by osteoblasts and potentially associated to the bone formation process, when compared to RR and RX counterparts [66,67]. Altogether, this information suggests that the absence of α-actinin-3 might predispose individuals to a higher likelihood of bone injuries, but this fact has not been reported yet in healthy or trained individuals.

**Figure 1:**
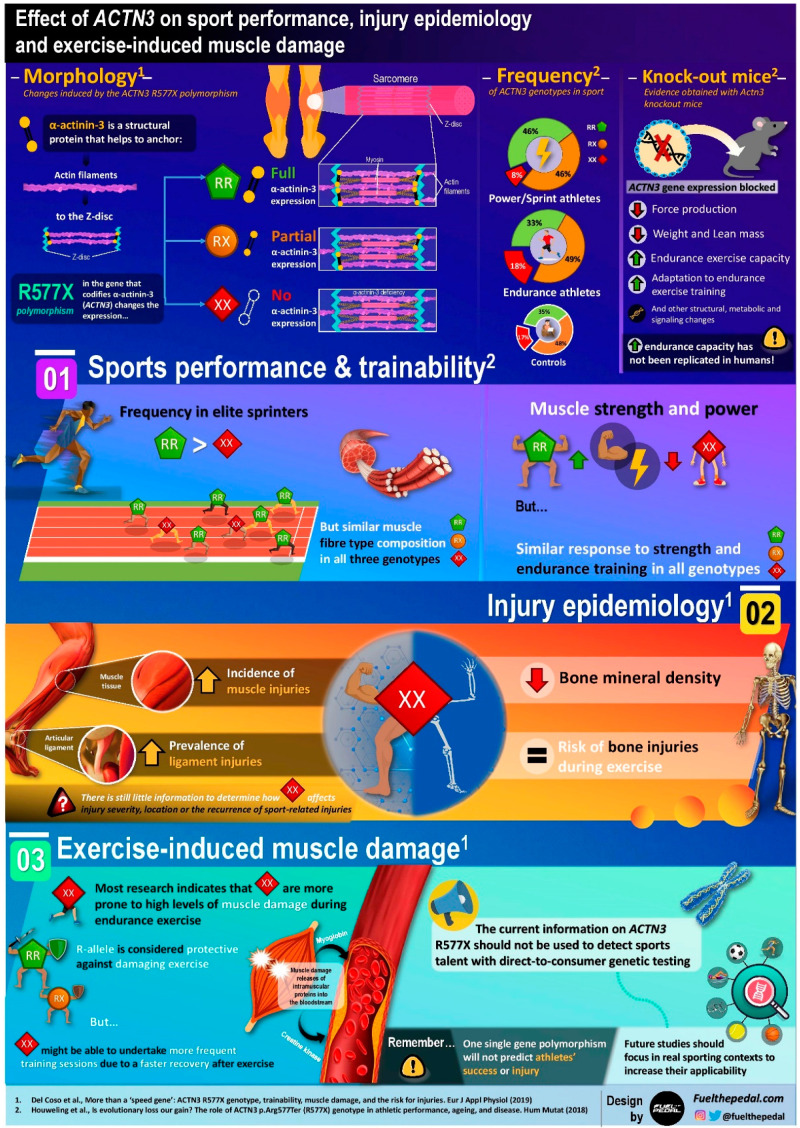
Infographic with a visual explanation of the different effects of the *ACTN3* genotype on sports performance, exercise-induced muscle damage, and injury epidemiology. α-actinin-3 is a protein located within the skeletal muscle with a key role in the production of sarcomeric force. A common stop-codon polymorphism (rs1815739; R577X) in the gene that codes for α-actinin-3 (*ACTN3*) produces individuals with the XX genotype that lack expression of functional α-actinin-3. In contrast, individuals with the R-allele (i.e., RX vs. RR genotypes) in this polymorphism can express α-actinin-3. Several investigations have found that α-actinin-3 deficiency due to XX homozygosity in the *ACTN3* R577X polymorphism can negatively affect sports performance through several structural, metabolic, or signaling changes. In addition, new evidences suggests that α-actinin-3 deficiency may also impact sports performance through indirect factors such a higher risk for injury or lower resistance to muscle-damaging exercise.

In summary, considering all the evidence presented above regarding the epidemiology of exercise-associated muscle injuries, there is some emerging evidence suggesting that α-actinin-3-deficient individuals may be more prone to suffer several types of injuries, namely muscle and ligament injuries (Figure 1). Additionally, it remains to be seen if the low bone mineral density displayed in XX women is also present in XX men, together with its potential effect on sport-related injuries. Nevertheless, the evidence is still scarce, mainly because to date, only a few investigations have compared the injury epidemiology in athletes with different *ACTN3* genotypes. 

## 5. Effect of α-Actinin-3 Deficiency on Exercise-Induced Muscle Damage

Damage to the skeletal muscle fiber produced by exercise activities involving high-intensity eccentric contractions, or a large volume of concentric contractions, particularly in muscle structures unaccustomed exercise, is known as exercise-induced muscle damage [68]. This is a physiological process that is typically accompanied by delayed onset of muscle soreness, local inflammation and swelling, and leakage of intramuscular proteins, such as creatine kinase (CK), myoglobin, and lactate dehydrogenase (LDH) into the blood. Exercise-induced muscle damage produces a mechanical disruption of the affected muscle fibers which in turn leads to pain and inflammatory response, both reducing the ability of the muscle to produce force. Although high levels of exercise-induced muscle damage has been mostly investigated from a clinical perspective because they may entail exertional rhabdomyolysis [69], the degree of muscle damage attained during exercise may be considered as a performance factor in some endurance-based disciplines [70].

In this regard, the absence of α-actinin-3 in muscle fibers also appears to limit their resistance to overcoming the load during eccentric and concentric muscle actions. This notion is based on several investigations indicating that X-allele carriers, particularly XX individuals, have higher levels of serum markers of muscle damage and higher self-reported values of muscle pain when compared to individuals possessing the R-allele [10]. Increased elevations in serum CK levels [71,72] and higher muscle pain values [71] have been observed in XX individuals after different eccentric exercise protocols when compared to their RR counterparts. These findings have been replicated in exercise activities that are known to result in exercise-induced muscle damage such as marathon running [73], half-triathlon races [74], and ultra-endurance adventure races [75]. This information shows that α-actinin-3-deficient individuals not only display increased levels of CK, but also experience higher reductions in muscle performance compared to R-allele carriers. The role of the *ACTN3* gene in the level of muscle damage during an endurance competition has been considered more important than the role of other genes with potential effects on the response to muscle-damaging exercise [76,77]. Still, there is some conflicting data with studies that found no association between the *ACTN3* genotype and increased exercise-induced muscle damage [78,79]. In fact, one investigation indicated that XX individuals might be able to undertake more frequent training sessions because they might recover faster after muscle-damaging exercise [80]. Therefore, it appears that the absence of α-actinin-3 produced by the *ACTN3* genotype could induce higher levels of muscle breakdown during certain exercise activities although it is necessary to determine if they also recover faster. This might be due to a possible structural benefit within the muscle fiber conferred by presence of the α-actinin-3 protein, which may help to produce a more resistant muscle fiber to resist the potential damage induced by high-intensity and endurance exercise. In this respect, α-actinin-3 would play a role during the eccentric phase of muscle contractions, conferring a higher capacity to skeletal muscle as a whole to resist muscle damage despite the restricted expression of this protein to fast type II muscle fibers. 

## 6. Conclusions

All this information suggests that α-actinin-3 deficiency due to XX homozygosity in the *ACTN3* R577X polymorphism produces a structural and physiological impairment in type II muscle fibers that can negatively affect sprint- and power-based sports performance, along with a lower capacity to resist muscle-damaging exercise. More investigation is still needed to clearly depict that XX athletes have an increased the likelihood of suffering muscle and ligament injuries during exercise and sport-related activities. Current evidence suggests that α-actinin-3 deficiency does not have any beneficial role in increasing endurance capacity, at least from the evidence found in case-control studies with elite endurance athletes and with genotype–phenotype investigations [53]. Future investigations on this topic should be carried out in real sporting contexts to increase their applicability to sports performance and elite athletes. Furthermore, investigations with an appropriate number of participants to increase the statistical power of the analysis is also necessary as this is one of the weaknesses of current evidence.

To date, the scientific information available about the *ACTN3* R577X polymorphism should not be used to detect sports talent with direct-to-consumer genetic testing nor to predict physiological responses to training, injury occurrence, or exercise-induced muscle damage. It should be noted that gene polymorphisms rarely act alone in terms of sports performance, as the set of complex multifactorial interactions among different genes and environmental factors are responsible for the influence of genetics on sports performance. Therefore, the presence or absence of one gene polymorphism will probably induce a low effect on sports performance. In this regard, future investigations with genome-wide association studies (GWAS) in athletes categorized as elite will be necessary to determine the potential role of genetics in sports performance. As the number of elite athletes within a sport discipline is reduced in each country, international collaboration will be a requirement to efficiently advance in this field. For the moment, a few genes have been consistently associated with elite athletic performance, but the evidence is perhaps weak to support the use genetic information to predict sports performance. Recently, the International Federation of Sports Medicine has suggested that feedback of genetic data to individuals is not recommended unless the accuracy and precision of prediction by genetic information is assured by replication and validation studies [81]. *ACTN3* is one of the genes with a higher amount of evidence to support its influence on sports performance and it will probably lead the future use of genetics to detect and predict sports performance. As highlighted in our visual summary, rather than simply focusing in the effect of α-actinin-3 deficiency due to the *ACTN3* XX genotype on “overall” sports performance, future research should seek to determine the effect of this genotype on trainability (for both strength and endurance-based training), on injury incidence and epidemiology, and on exercise-induced muscle damage. More investigations, and replication of findings, are necessary to validate the use of genetics to unquestionably predict performance or any other physical or physiological outcomes that can affect athletic performance.
